# Alleviation Effects of GQD, a Traditional Chinese Medicine Formula, on Diabetes Rats Linked to Modulation of the Gut Microbiome

**DOI:** 10.3389/fcimb.2021.740236

**Published:** 2021-10-08

**Authors:** Jiaxing Tian, Bingbing Bai, Zezheng Gao, Yingying Yang, Haoran Wu, Xinmiao Wang, Jun Wang, Min Li, Xiaolin Tong

**Affiliations:** ^1^ Department of Endocrinology, Guang’anmen Hospital, China Academy of Chinese Medical Sciences, Beijing, China; ^2^ CAS Key Laboratory of Pathogenic Microbiology and Immunology, Institute of Microbiology, Chinese Academy of Science, Beijing, China

**Keywords:** gut microbiota, type 2 diabetes mellitus, Chinese herbal medicine, intestinal barrier function, inflammation

## Abstract

Gegen Qinlian Decoction (GQD) is a Chinese herbal medicine that has been reported to significantly decrease blood glucose levels, which is suggested to be related to interactions with the gut microbiota. However, the protective effect of GQD on intestinal barrier function with regard to its influence on the gut microbiota has not been explored to date. In this study, we investigated the role of the gut microbiota in mediating the hypoglycemic mechanism of GQD in type 2 diabetes mellitus (T2DM) rats induced by a single intraperitoneal injection of streptozotocin after 4 weeks of high-fat diet feeding. The T2DM rats were randomly allocated to receive GQD, metformin (Met), or saline for 12 consecutive weeks, and changes in metabolic parameters, intestinal barrier function, and inflammation were investigated. Gut microbiota was analyzed using 16S rRNA gene sequencing from fecal samples, and statistical analyses were performed to correlate microbiota composition with phenotypes of the T2DM rats. GQD administration decreased the levels of blood glucose and inflammatory cytokines, and increased the levels of tight junction proteins. Besides, GQD had a protective effect on islet function, restoring intestinal permeability, and inhibiting inflammation, as evidenced by increases in the levels of serum C-peptide, occludin, and claudin-1 in the colon, and also improved the expression of serum inflammatory factors. In addition, GQD regulated the structure of the gut microbiota by increasing the proportions of short-chain fatty acids-producing and anti-inflammatory bacteria, and decreasing the proportions of conditioned pathogenic bacteria associated with the diabetic phenotype. Overall, these findings suggest that GQD could ameliorate hyperglycemia and protect islet function by regulating the structure of the gut microbiota, thereby restoring intestinal permeability and inhibiting inflammation in T2DM rats. Our study thus suggests that the hypoglycemic mechanism of GQD is mediated by its modulation of the gut microbiota.

## 1 Introduction

Gegen Qinlian Decoction (GQD), a classical prescription of traditional Chinese medicine, was first recorded in the book “Treatise on Febrile Diseases” in the Eastern Han Dynasty. The GQD consists four Chinese herbs, namely *Radix Puerariae, Radix Scutellariae, Rhizoma Coptidis, and Radix Glycyrrhizae*. GQD has been widely used to treat gastrointestinal diseases such as enteritis and bacillary dysentery, demonstrating its treatment principle of relieving the exterior pathogen and clearing the interior heat in TCM theory. Moreover, GQD has been reported to show a potent hypoglycemic effect, and has thus also been used in the clinical treatment of patients with diabetes (Zha et al., 2015; Cao et al., 2021; China Diabetes Society, 2021). These effects have been shown to be achieved by various mechanisms, including ameliorating the insulin signaling pathway, improving circulation, inhibiting inflammation, and regulating metabolomics and immunity (Shan et al., 2017; Zhang et al., 2017; Du et al., 2018; Yuan et al., 2018; Zhou et al., 2018).

The critical role of the gut microbiota in metabolic diseases has been increasingly recognized in recent years (Boulangé et al., 2016; Wu et al., 2017). Gut microbiota mediate the physiological processes involved in the biotransformation and synthesis of biologically active small molecules, and form a dynamic balance between the human body and the environment (Sonnenburg and Bäckhed, 2016; Singh et al., 2017). However, risk factors for metabolic disorders such as lack of exercise and irregular eating habits result in dysfunction of the gut microbiota and turbulence of the internal environment with consequent destruction of the integrity of the intestinal barrier, allowing for the proliferation of opportunistic pathogens and pathogenic bacteria (Forslund et al., 2015). These effects induce chronic inflammatory reactions that disrupt metabolic balance (Cani et al., 2008; Cani and Delzenne, 2009). Indeed, some hypoglycemic drugs have been shown to function by interfering with the structure of the gut microbiota, such as metformin (Met) and acarbose (Gu et al., 2017; Wu et al., 2017).

Similar to these typical drugs, we previously found that GQD could decrease blood glucose levels by enriching the amounts of beneficial bacteria, and changes in the gut microbiota structure were associated with the anti-diabetes effects of GQD (Xu et al., 2015). In addition, berberine, one of the main active components of GQD, was found to modulate the gut microbiota by enriching the short-chain fatty acids (SCFAs)-producing bacteria and reducing the microbial diversity, which contributed to its beneficial effects on host metabolism (Zhang et al., 2015). However, no study has explored the influence of the gut microbiota with respect to the protective effect of GQD on intestinal barrier function, which may provide insight into the mechanism of GQD in alleviating type 2 diabetes mellitus (T2DM) from another perspective.

Therefore, the aim of the present study was to investigate the influence of GQD on the gut microbiota, intestinal barrier function, and inflammation in the common diabetic model induced with injection of streptozotocin (STZ, a toxic glucose analogue which can destroy the beta cells) (Lenzen, 2008) after 4 weeks of high-fat diet (HFD) feeding (Gheibi et al., 2017). We used 16S rRNA sequencing from fecal samples to analyze the gut microbiota of T2DM rats, and conducted correlation analysis between aspects of the diabetic phenotype and gut microbiota toward further elucidating the possible mechanism of islet function protection by GQD.

## 2 Materials and Methods

### 2.1 GQD Preparation

GQD consists of four herbs, including *Radix Puerariae*, *Radix Scutellariae*, *Rhizoma Coptidis*, and *Radix Glycyrrhizae*, which was produced at the Department of Pharmacy of Guang’anmen Hospital (Beijing, China). The raw herbs were provided from Sichuan New Green Pharmaceutical Technology Co. LTD., and the phytochemical profiles of the GQD were confirmed with high-performance liquid chromatography as described previously (Xu et al., 2015). For preparation of GQD, the herbs were first soaked in 10 times of the materials weight of water for 1 hour and then boiled two times, 1.5 h for the first time and 1 h for the second time. After filtration, the decoction was concentrated, and then packaged and stored at 4°C for further use.

### 2.2 Animals and Experimental Design

**Figure 1:**
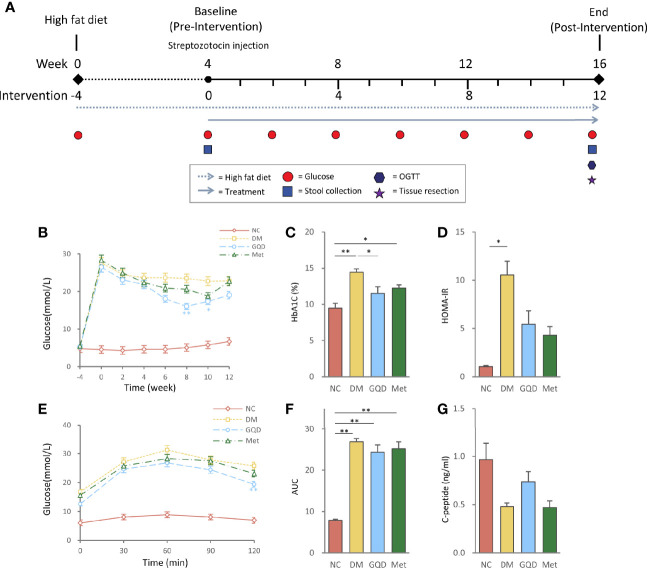
Intervention of GQD decreased blood glucose, and improved oral glucose tolerance and insulin resistance status. **(A)** Flow diagram of the experimental design. **(B)** Blood glucose levels over the 12-week experiment. **(C)** HbA1c levels determined after treatment for 12 weeks. **(D)** HOMA-IR index was calculated using the following formula: HOMA-IR= FINS × FPG/22.5. **(E)** Oral glucose tolerance test. **(F)** AUC calculated from the glucose concentrations versus time for the period of 0-120 min after glucose load by the trapezoidal rule. **(G)** Serum C-peptide levels determined after treatment for 12 weeks. Values are expressed as means ± SE. Differences were assessed by ANOVA: **p < 0.01, *p < 0.05. HbA1c, glycosylated hemoglobin; HOMA-IR, homeostasis model assessment for insulin resistance; FINS, fasting insulin; FPG, fasting plasma glucose; NC, normal control; DM, diabetes mellitus; GQD, Gegen Qinlian Decoction; Met, metformin.

The experiment was approved by the Local Ethics Committee for Animal Research Studies at Guang’anmen Hospital. All methods were performed in accordance with relevant guidelines and regulations. Adult male Wistar rats (Beijing Vital River Laboratory Animal Technology Co., Ltd.), weighing 190–210 g, were kept in a specific pathogen-free laboratory with free access to water throughout the experiment. The rats were housed in a restricted-access room with controlled temperature (22–25°C) and light/dark (12 h/12 h) cycle. DM was induced in 18 rats by a single intraperitoneal injection of 30 mg/kg STZ (S0130, Sigma-Aldrich Co. LLC, MO, USA) after high fat diet (HFD) feeding (D12492; Research Diets, Inc., NJ, USA) for 4 weeks, which resulted in a random blood glucose level ≥11.1 mmoL/L 72 h after the injection. These T2DM rats were then randomly subdivided into the following three groups (n = 6 per group): DM group, GQD group, and Met group. Another six Wistar rats of similar age and body weight were used as the normal control group (NC). The rats were administered 25 g/kg GQD [based on previous study and relevant pharmacological data (Wei et al., 2010; Xu et al., 2015)], 250 mg/kg Met (Sino-American Shanghai Squibb Pharmaceuticals Ltd., Shanghai, China), or distilled water in the volume equivalent to GQD (DM and NC groups) once daily by gavage, which passed though the esophagus and directly reached the stomach lumen, for 12 weeks. At the end of the experiment, the rats were fasted overnight and anesthetized with 10% chloral hydrate (3.5 mL/kg, intraperitoneal injection; 30037516, Sinopharm Chemical Reagent Co., Ltd., Beijing, China), and then subjected to glycometabolism examinations, evaluation of intestinal barrier function, and expression of serum inflammatory factors. The timepoints of interventions and examinations were shown in **Figure 1A**.

### 2.3 Glycometabolism Examinations

The blood glucose levels of the rats were monitored every 2 weeks after initiating the experiment. One drop of blood was obtained from the tail vein, and blood glucose was measured by a OneTouch glucometer (Roche Ltd., Basel, Switzerland). For glycosylated hemoglobin (HbA1c), C-peptide, and insulin measurements, blood was obtained from the abdominal aorta at the end of the experiment, and serum was separated for C-peptide and insulin measurements. HbA1c kit was purchased from Roche Ltd (Tina-quant Hemoglobin A1c Gen.2, Basel, Switzerland). C-peptide kit and insulin kit were purchased from Elabscience Biotechnology Co., Ltd (E-EL-R0032c; E-EL-R2466c, Wuhan, China). HbA1c was measured by immunoturbidimetry (AU480, Japan). Serum C-peptide and insulin were measured by enzyme-linked immunosorbent assay (ELISA; Multiskan MK3, Thermo, USA). All the examinations were conducted according to the manufacturer’s instructions. The oral glucose tolerance test (OGTT) was performed 2–3 days before sacrifice. After 12 h of fasting, the glucose solution (2 g/kg) was administered by oral gavage. Blood glucose levels at time 0 (fasting glucose, taken before glucose gavage) and at 30, 60, 90, and 120 min after glucose gavage were analyzed using the OneTouch glucometer (Roche). The glucose level was then plotted against time, and areas under the glucose curve (AUC) of the glucose concentration versus time for the period of 0-120 min after glucose infusion were calculated by the trapezoidal rule. Homeostasis model assessment for insulin resistance (HOMA-IR) = [Insulin (μmol/l) × Glucose (mmol/l)/22.5] and homeostasis model assessment for β-cell function (HOMA-β) = [20 × Insulin (μmol/l)]/[Glucose (mmol/l) - 3.5] were calculated as described previously (Matthews et al., 1985).

### 2.4 Intestinal Barrier Function

The jejunum, ileum, and colon were harvested after sacrifice and frozen at −80°C. Total RNA was isolated from the rat intestinal tissues with TRIzol reagent (15596026, Invitrogen, Carlsbad, CA, USA) and subjected to quantitative polymerase chain reaction (qPCR) with SYBR FAST qPCR Kit Master Mix (2×) Universal (KAPA Biosystems, USA). The thermal cycling conditions were as follows: 95°C for 10 min, 45 cycles at 95°C for 10 s and 59°C for 60 s, followed by 95°C for 15 s, 72°C for 15 s, and 95°C for 15 s. The mRNA expression levels of zonula occludens-1 (ZO-1), occludin, and claudin-1 were quantitatively analyzed and normalized to *Gapdh* levels. The forward and reverse primer sequences were as follows: ZO-1 forward 5′-GAGCAAGCCTCCTGCACATA-3′, reverse 5′- TCAGTTTCGGGTTTCCCCTT-3′; occludin forward 5′-CAACGGCAAAGTGAATGGCA-3′, reverse 5′-CTTTCCCCTTCGTGGGAGTC-3′; claudin-1 forward 5′-TGGGGACAACATCGTGACTG-3′, reverse 5′-CCCCAGCAGGATGCCAATTA-3′; *Gapdh* forward 5′-TGTGAACGGATTTGGCCGTA-3′, reverse 5′-GATGGTGATGGGTTTCCCGT-3′.

### 2.5 Serum Cytokine/Protein Levels

For serum cytokine and protein measurements, blood was obtained from the abdominal aorta at the end of the experiment, and serum was separated. C-reactive protein (CRP) kit and adiponectin kit were purchased from Elabscience Biotechnology Co., Ltd (E-CL-R0021c, Wuhan, China). Interleukin-1β (IL) -1β kit, tumor necrosis factor-α (TNF)-α kit and monocyte chemoattractant protein-1 (MCP-1) kit were purchased from Neobioscience Biotechnology Co., Ltd (ERC007; ERC102a; ERC113, Shenzhen, China), and the endotoxins were obtained from BG Biotechnology Co., Ltd (Shanghai, China). The CRP, IL-1β, TNF-α, MCP-1, endotoxins, and adiponectin were measured by ELISA (Multiskan MK3, Thermo, USA). All the test followed the manufacturer’s instructions.

### 2.6 16S rRNA Gene Analysis

Stool samples from each rat were collected before and after the intervention. The 16S rRNA metagenomic sequencing libraries were prepared according to the manufacturer’s instructions (Illumina). The data was randomly subsampled to 10000 reads. The gene-specific sequences used in our study targeted the 16S V3 and V4 regions. Each 16S library was sequenced in a separate 250-bp, paired-end run on the Illumina Hiseq 2500 platform. Sequence analysis was performed using HiSeq SBS Kit V4 following the detailed methods described in our previous study (Falony et al., 2016). Fast Length Adjustment of Short reads (FLASH) was used to merge paired-end reads from next-generation sequencing (Magoč and Salzberg, 2011). Low-quality reads were filtered by fastq_quality_filter in FASTX Toolkit (http://hannonlab.cshl.edu/fastx_toolkit/). Chimeras were removed using the USEARCH program’s UCHIME command and the ‘GOLD’ database. We only chose the genera with average abundance more than 1% into analysis. The taxonomical classification of reads was determined using the RDP classifier to generate composition matrices at the genus to phylum levels (Wang et al., 2007). A bootstrap value > 0.8 was considered as high-confidence taxonomy assignment, while low-confidence sequences were labeled as unclassified assignment. All the 16 rRNA gene sequencing data were deposited in the Genome Sequence Archive database (see “Data Availability” part).

### 2.7 Statistical Analysis

Data are presented as mean ± standard errors of the mean. One-way analysis of variance (ANOVA) or Wilcoxon rank-sum test was used for comparisons among groups in SPSS19 software (SPSS Inc., Chicago, IL, USA). Alpha and beta diversities were analyzed. The number of genera and Shannon index were calculated to identify community richness and diversity. For beta diversity, principal coordinates analysis (PCoA) was applied to examine dissimilarities in community composition and microbiota abundances. In addition, linear discriminant analysis of effect size (LEfSe) (Segata et al., 2011) was performed to calculate taxon abundance and to determine the differences among groups (linear discriminant analysis score > 2 and *P* < 0.05 were considered significant). The correlation between diabetic phenotype and gut microbiota composition was assessed using Pearson correlation coefficients. P < 0.05 was considered statistically significant.

## 3 Results

### 3.1 GQD Improves Metabolic Disorder and Inflammation

#### 3.1.1 Metabolic Parameters

There were no significant differences in baseline variables among the four groups of rats prior to the intervention. After 12 weeks of treatment, both Gegen Qinlian Decoction (GQD) and metformin improved blood glucose, HbA1c, and HOMA-IR levels. However, only GQD significantly decreased fasting plasma glucose at 8 and 10 weeks, and HbA1c at 12 weeks (**Figures 1B, C**, *P* < 0.01, *P* < 0.05), although no significant difference was observed between the GQD and Met groups based on the ANOVA results. There were no significant differences among treatment groups and DM groups (**Figure 1D**). Compared with the DM group, both GQD and Met improved the glucose level and AUC of the OGTT, with no significant differences between the two treatment groups, except for a significant decrease in the fasting plasma glucose level at 120 min in the GQD group (**Figures 1E, F**, *P* < 0.01). GQD also resulted in a slight increase of C-peptide level compared with the DM and Met groups, although the differences were not statistically significant according to ANOVA (**Figure 1G**).

#### 3.1.2 Intestinal Barrier Function

**Figure 2:**
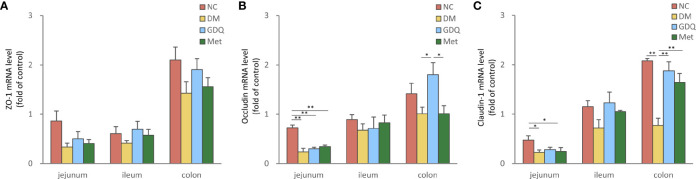
GQD alleviated the intestinal barrier function of T2DM rats. Expression levels of **(A)** ZO-1, **(B)** occludin, and **(C)** claudin-1 in the jejunum, ileum, and colon after treatment for 12 weeks. Values are expressed as means ± SE. Differences were assessed by ANOVA: **p < 0.01, *p < 0.05. ZO-1: zonula occludens-1. The fold over control means the fold change differences relative to DM based on 2(-delta delta CT) score.

Zonula occludens-1 (ZO-1), occludin, and claudin-1 are important integral membrane proteins which participate in tight junctions structural integrity to form the intestinal mucosal barrier (Furuse et al., 1994; Chen et al., 2020). The mRNA levels of ZO-1, occludin, and claudin-1 expressed in the intestine decreased in the DM group, indicating that diabetes induction with STZ destroyed the intestinal barrier function. After 12 weeks of treatment, both GQD and Met slightly elevated the expression levels of ZO-1 in the whole intestine (**Figure 2A**) and significantly increased the expression level claudin-1 in the colon (**Figure 2C**, *P* < 0.01). Compared with the DM and Met groups, GQD significantly increased the occludin expression level in the colon (**Figure 2B**, *P* < 0.05). These results indicated that GQD has superior effects at alleviating the DM-disrupted intestinal barrier function than Met.

#### 3.1.3 Inflammation

**Figure 3:**
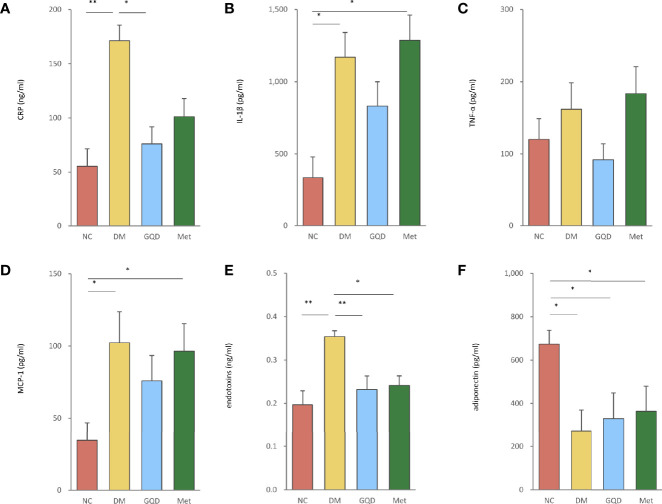
GQD inhibited inflammation of T2DM rats by regulating the levels of serum inflammatory factors. The serum levels of **(A)** CRP, **(B)** IL-1β, **(C)** TNF-α, **(D)** MCP-1, **(E)** endotoxins, and **(F)** adiponectin after treatment for 12 weeks. Values are expressed as means ± SE. Differences were assessed by ANOVA: **p < 0.01, *p < 0.05. CRP, C-reactive protein; IL-1β, interleukin-1β; TNF-α, tumor necrosis factor-α; MCP-1, monocyte chemoattractant protein-1.

GQD treatment reduced the elevated expression levels of CRP, IL-1β, TNF-α, MCP-1, and endotoxins of T2DM rats (**Figures 3A–E**). These parameters were also ameliorated by Met treatment, except for IL-1β and TNF-α levels (**Figures 3B, C**). However, only GQD significantly decreased both CRP and endotoxins (**Figures 3A, E**, *P* < 0.01, *P* < 0.05). We also found that metformin decreased the expression of endotoxins (**Figure 3E**, *P* < 0.05). GQD also showed improved alleviation of inflammation compared with the DM and Met groups. Compared with the NC group, DM, GQD, and Met all showed decreased level of adiponectin, however, both GQD and Met minorly improved the adiponectin level with no significant differences between the two treatment groups (**Figure 3F**).

### 3.2 GQD Alters the Gut Microbiota Structure

**Figure 4:**
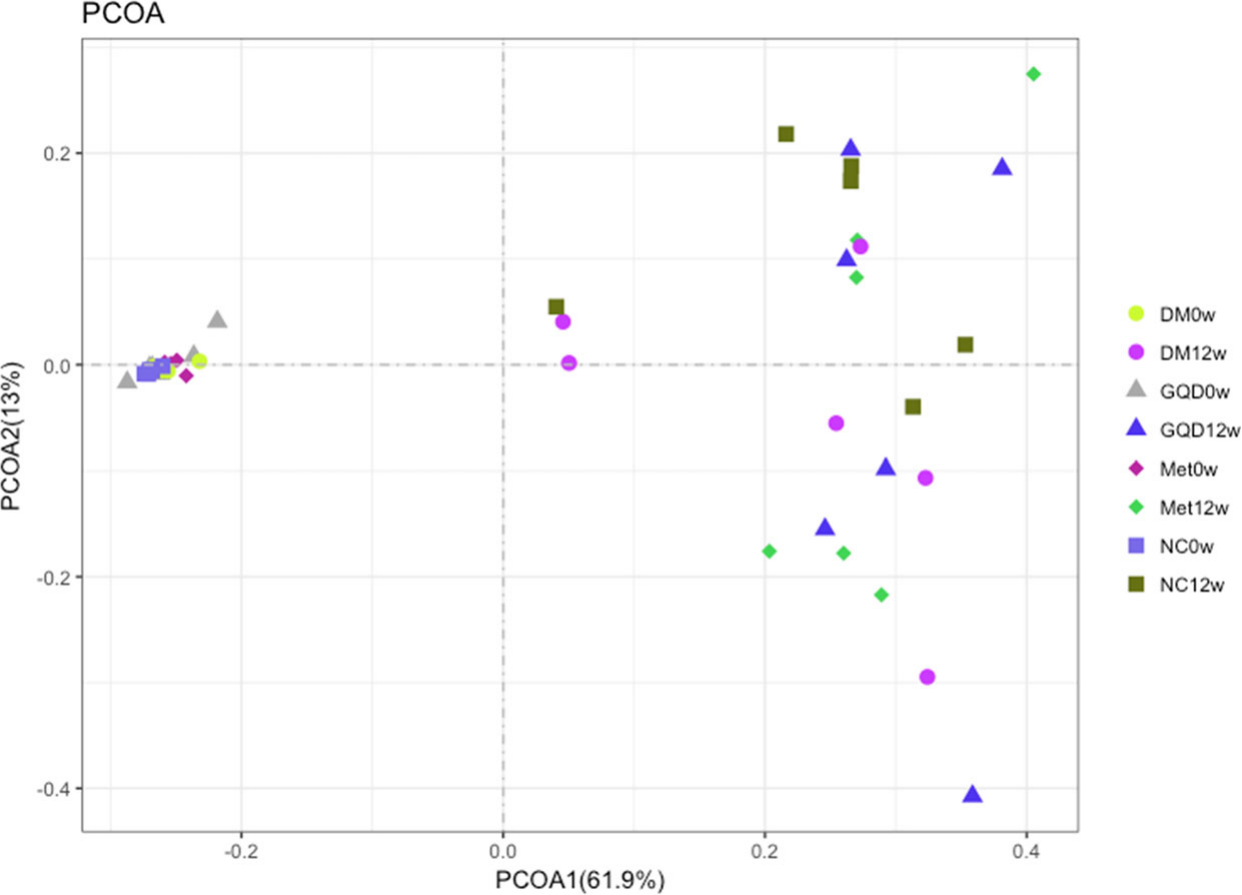
Comparison of the gut microbiota structure among different groups. Bray-Curtis distance PCoA of the rat fecal microbiota before and after different treatments.

The median coverage was 217680.5 reads in NC (range 108074-288659), 182302.5 reads of DM (range 43516-334672), 170758.5 reads of GQD (range 72308-213682), and 217035.5 reads of Met (range 104794-285379). Analysis of the diversity of the gut microbiota community based on 16S rRNA sequencing of the V3 and V4 regions at 0 and 12 weeks showed that both GQD and Met decreased the number of genera, although no significant difference was obtained (**Figure S1A** in the supplemental material). However, both GQD and Met significantly increased the Shannon index (P < 0.01), indicating that the diversity of the gut microbiota increased after treatment (**Figure S1B** in the supplemental material). PCoA of the microbiota composition showed that the four groups were clustered together before treatment and were clearly separated after 12 weeks of treatment. Thus, both GQD and Met changed the gut microbiota structure of T2DM rats (**Figure 4**).

**Figure 5:**
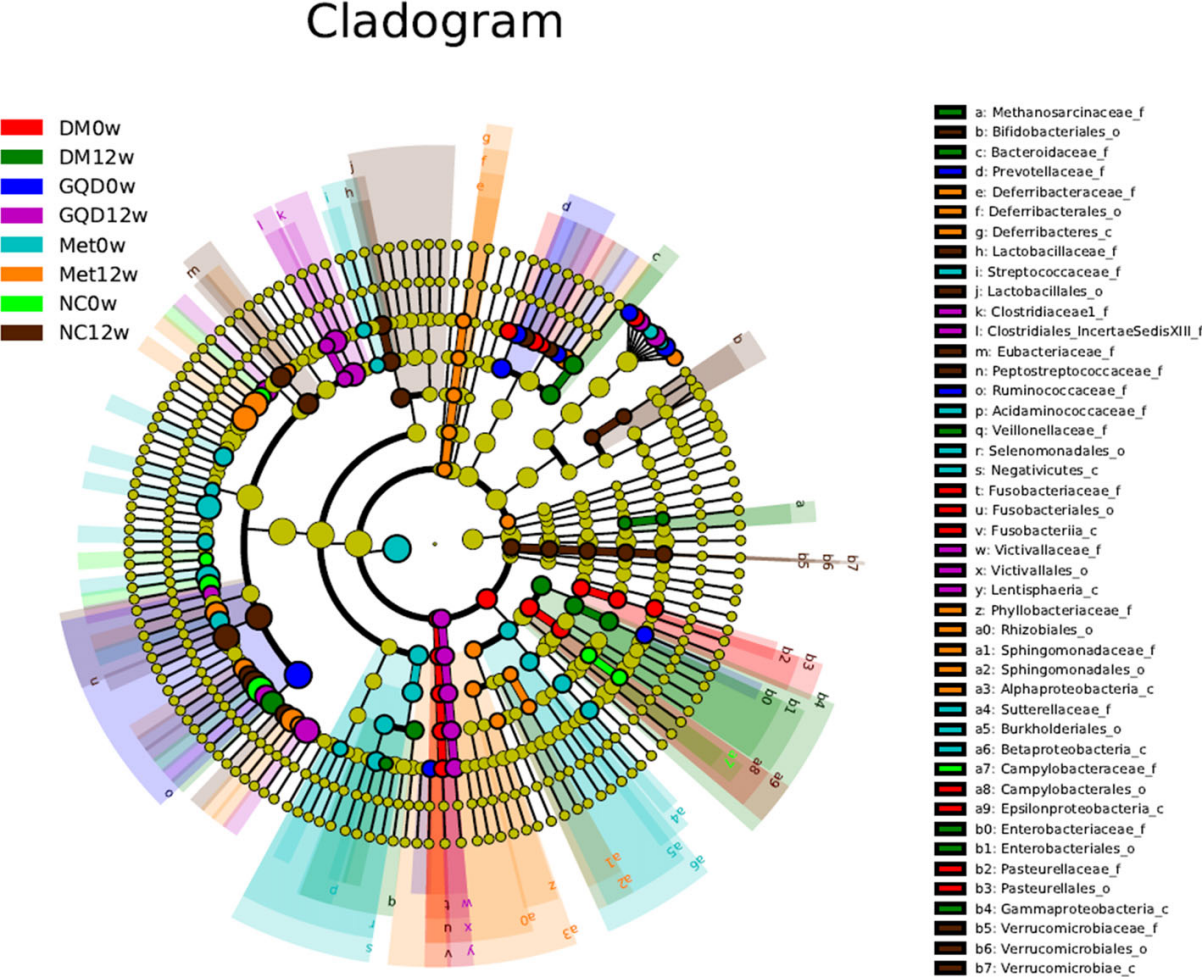
Taxonomic differences of gut microbiota among different groups. Taxonomic cladogram obtained by LEfSe before and after treatments. Differences are represented by the color of the most abundant class. The diameter of each circle is proportional to the taxon’s abundance.

To further investigate specific differences in the microbiota among groups, we used LEfSe to identify the altered bacterial phenotypes. The cladogram in **Figure 5** shows the dominant bacteria identified in each group. A total of 187 bacteria changed significantly among the GQD, Met, and DM groups, with a linear discriminant analysis score (log 10) > 3 (**Figure S2**). Constitutions of gut bacterial showed apparent variation at genus level among groups. After 12 weeks of treatment, the main differential microbial lineages of the T2DM model rats included increased abundances of *Peptostreptococaceae*, *Romboutsia*, *Ruminococcus*, and *ClostridiumIV*, and decreased abundances of *Faecalibacterium*, *unclassified_Lachnospiraceae*, *Ruminococcus2*, *Roseburia*, and *Gemmiger*. The abundance of *unclassified_Christensenellaceae* increased in all groups except for the NC group, and the abundances of *Blautia* and *Romboutsia* increased in all groups except for the DM group.

The changes in the composition of functional microbiota also clearly differed among groups after treatment (**Figure S2**). Compared with the NC, the DM grouped showed a decreased level of beneficial bacteria such as Lactobacillales (including *Lactobacillaceae* and *Lactobacillus*) and bacilli. At the same time, opportunistic pathogens such as *ClostridumXI* and *ClostridiumXIV* were enriched in the DM group, reflecting a state of gut microbiota dysbiosis. Compared with the DM group, the levels of beneficial bacteria such as *Flavonifractor* and *Acetatifactor* increased, whereas those of opportunistic pathogens such as *Butyricimonas*, *Anaerofustis*, *Butyricicoccus*, and *Gammaproteobacteria* decreased in the GQD group. Similarly, in the Met treatment group, *Oscillibacter*, *Flavonifractor*, *Alphaproteobacteria*, and *Anaerotruncus* increased, while the abundances of *Sutterellaceae*, *Parasutterella*, *Terrisporobacter*, and *Coprococcus* decreased compared with those of the DM group. Comparison of the key altered bacterial phenotypes that responded to GQD and Met showed that *ClostridiumXI*, *Anaerostipes*, and *Gammaproteobacteria* were enriched by Met. In addition, *Flavonifractor* increased in both the GQD and Met groups, indicating a common bacterial phenotype, which might be related to their mechanism of action in alleviating diabetes rather than simply a direct effect of the drug.

### 3.3 Correlation of Gut Microbiota With Biochemical Parameters

**Figure 6:**
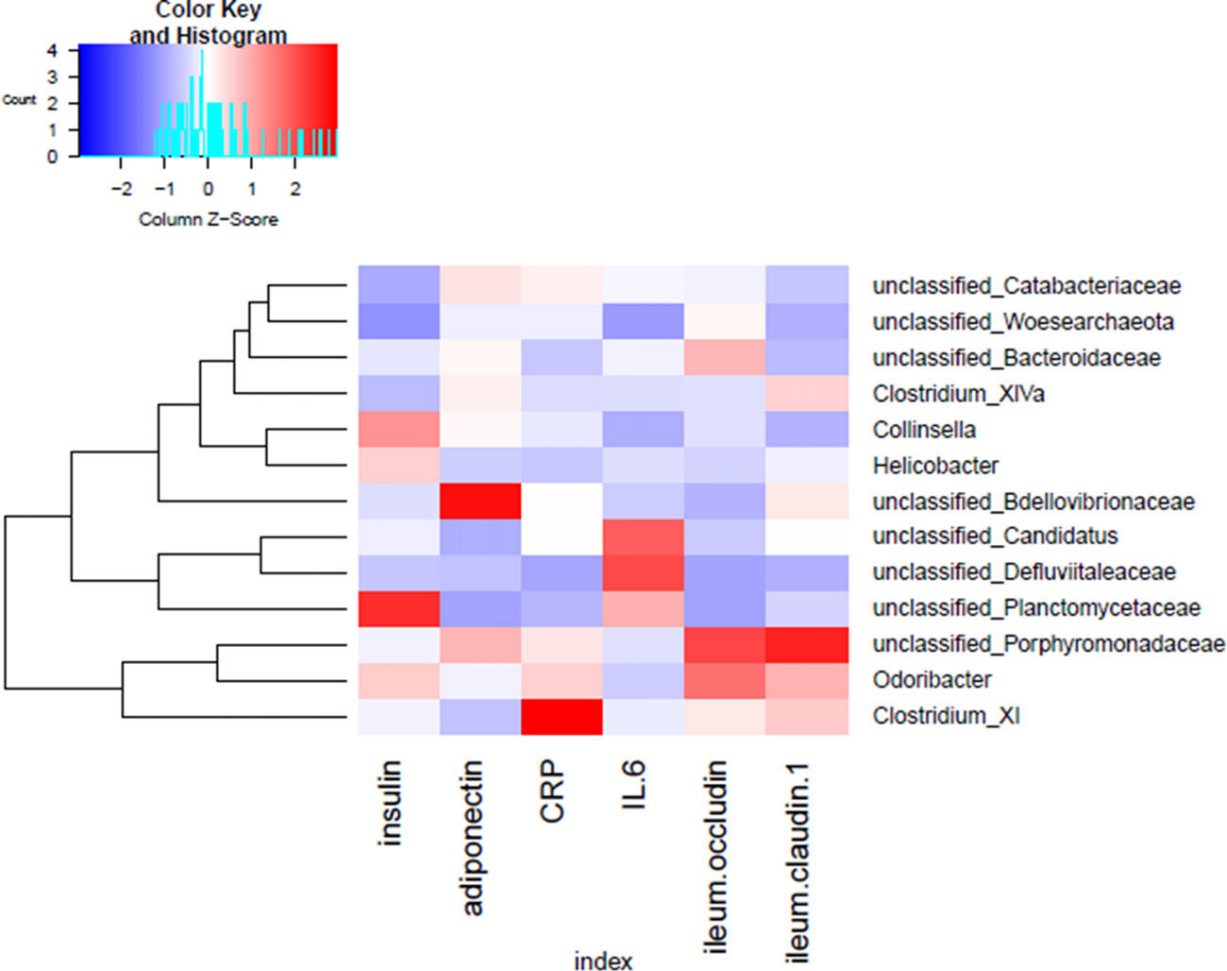
Correlation analysis between the gut microbiota and T2DM-related phenotype in rats. Heatmap of correlation between the alterations in the gut microbial population and changes in host parameters related to insulin, inflammatory factors, and intestinal barrier function. Pearson correlation values were used for generation of the matrix.

To identify key phylotypes correlated with the therapeutic efficacy of GQD and Met, correlation heatmap analysis was performed between the identified genera of the gut microbiota and T2DM-related biochemical parameters (insulin, adiponectin, CRP, IL-6, occludin, and claudin-1 in the ileum). As shown in **Figure 6**, unclassified *Planctomycetaceae* was positively correlated with insulin, while unclassified *Bdellovibrionaceae* was positively associated with adiponectin. Moreover, *Clostridium XI* was positively correlated with the inflammation marker CRP, while *Odoribacter* and unclassified *Porphyromonadaceae* were positively associated with intestinal barrier function markers (occludin and claudin-1 in the ileum). Thus, the change in gut microbiota dysbiosis may be associated with biochemical parameters.

## 4 Discussion

Our previous study demonstrated that GQD could decrease the blood glucose level and enrich the amounts of beneficial bacteria, and these structural changes of gut microbiota were associated with the anti-diabetes effects of GQD (Xu et al., 2015). Consistently, in the present study, GQD lowered the blood glucose level in T2DM model rats, and improved the status of insulin resistance. We further confirmed that GQD has a stronger hypoglycemic effect than Met, and increased the levels of C-peptide, demonstrating a protective role in islet function.

Intestinal barrier function and the expression of tight junction protein both significantly decrease in a condition of metabolic disturbance (Cani et al., 2009). Oliveira et al. (2019) found that exposure to the small intestine luminal content isolated from HFD-fed mice induced a more significant decrease in transepithelial electrical resistance, accompanied by a significant decrease in the levels of the tight junction proteins claudins, occludin, and ZO-1. In addition, Genser et al. (2018) found tight junction impairments in the jejunal epithelium of obese patients. Serum levels of zonulin and lipopolysaccharide (LPS)-binding protein, two markers of intestinal barrier alterations, were also increased in the obese patients. Our present results showed decreased levels or decreased tendency of ZO-1, occludin, and claudin-1 in the DM group, indicative of disruption of the tight junction barrier under diabetes. Although both GQD and Met increased the levels of these tight junction proteins, GQD showed better potential at restoring intestinal permeability owing to the greater increases in occludin and claudin-1 levels in the colon.

This loss of control of intestinal permeability is accompanied by increased serum levels of endotoxins (Creely et al., 2007; de Kort et al., 2011; Genser et al., 2018). Moreover, high-fat and high-caloric diets can favor the intestinal colonization of gram-negative microbiota, leading to increased plasma LPS levels (metabolic endotoxemia) (Wang J. et al., 2017). This combination of increases in intestinal permeability and plasma LPS levels induce the production of pro-inflammatory cytokines (ILs, TNF-α) and CRP that can in turn induce the serine phosphorylation of insulin receptor substrate-1, leading to insulin resistance (Hotamisligil, 2006; Hotamisligil and Erbay, 2008). Since GQD significantly decreased the levels of inflammatory factors, especially endotoxin and CRP, and showed more effective reduction of IL-1β, TNF-α, and MCP-1 than Met, it has good potential to protect against the progress of DM. Moreover, both GQD and Met increased the levels of adiponectin. Yamauchi et al. (2001) suggested that adiponectin supplementation could modify the insulin resistance. Thus, GQD appears to alleviate insulin resistance by inhibiting inflammation and strengthening intestinal barrier function.

Moreover, the ability of GQD to restore intestinal permeability *via* increasing occludin and claudin-1 was found to be closely related to changes in the gut microbiota. Indeed, the gut microbiota was shown to be crucial for the efficient development and maintenance of the intestinal barrier (Alam and Neish, 2018). Thus, destabilized equilibrium between the gut microbiota and the host could lead to a wide range of diseases characterized by the disrupted intestinal barrier (Hamilton et al., 2015). Conversely, the microenvironmental changes in the injured mucosa favor a pro-regenerating consortium of bacteria that promote mucosal wound repair and restoration of barrier functions (Wang et al., 2018).

Consistent with previous studies (Chen et al., 2018; Tong et al., 2018), the richness of gut microbiota decreased after GQD administration, which could be related to the antibacterial effect of berberine, an effective component of GQD (Zou et al., 2017). Moreover, the DM group had lower levels of beneficial bacteria compared with the NC group, such as *Lactobacillales*, which could significantly delay the onset of hyperglycemia and dyslipidemia (Yadav et al., 2007; Andreasen et al., 2010; Ejtahed et al., 2011). Along with the loss of these beneficial bacteria, opportunistic pathogens such as *ClostridumXI* and *ClostridiumXIV* were enriched in the DM group. An *in vitro* study suggested that flagellated *Clostridium XIV* bacteria contribute to the development of obesity through distorted adipose tissue metabolism and inflammation (Pekkala et al., 2015). Moreover, the abundance of *Clostridium XI* was positively correlated with the inflammation index. At the same time, *Odoribacter* was positively correlated with intestinal barrier function indexes (occluding and claudin-1 in the tissue of ileum) and declined in Met-exposed offspring of HFD-fed mice (Salomäki-Myftari et al., 2016). Although the relationship between the gut microbiota and intestinal barrier function requires further investigation, these previous studies and our results suggest that the decrease of SCFAs-producing bacteria and an increase of conditioned pathogens in DM could be modified by GQD treatment to restore balance.

Although both GQD and Met changed the structure of the gut microbiota in T2DM rats, the specific taxonomic groups affected were distinct. GQD largely increased the levels of beneficial bacteria such as *Flavonifractor* and *Acetatifactor*, and decreased opportunistic pathogenic bacteria such as *Anaerofustis* and *Gammaproteobacteria*. The abundance of *Flavonifractor* was correlated with high levels of SCFAs (Borrelli et al., 2017) and negatively correlated with body mass index in previous studies (Kasai et al., 2015; Borgo et al., 2018). *Acetatifactor*, identified as a bile acid-induced anaerobic bacterium (Pfeiffer et al., 2012), produces SCFAs such as acetate and butyrate (Pathak et al., 2018). SCFAs could improve the clinical features of T2DM (Puddu et al., 2014), including decreasing the serum levels of glucose, insulin resistance, and inflammation (den Besten et al., 2013; Morrison and Preston, 2016). Patrone et al. found increased abundance of *Anaerofustis* in high-saturated-fat diet-fed mice, which was positively correlated with cholesterol and triglycerides levels (Patrone et al., 2018). However, *Anaerofustis* was also found to be positively correlated with the fecal concentration of dimethylamine, which reduced in rats fed a lard-based high-fat diet (Lin et al., 2016). Pindjakova et al. showed that *Gammaproteobacteria* was more abundant in obese mice (Pindjakova et al., 2017), while the Roux-en-Y gastric bypass (RYGB) could also result in increases in *Gammaproteobacteria* (Guo et al., 2017).

The normal feed for the normal group was consistent with the feed used for breeding the rats. Though there was a small change in the structure of the gut microbiota in the normal group in this study and there may be some effect correlated with time or feed; but our study adhered to the same feed to minimize the effect of the intervention and the basic flora structure has not change.

In conclusion, our study demonstrates that GQD could improve the hyperglycemia, intestinal barrier function, and inflammation, while restoring the dysregulated gut microbiota in T2DM rats to reach a normal condition. Moreover, GQD treatment specifically increased the abundance of SCFAs-producing and anti-inflammatory bacteria such as *Flavonifractor* and *Acetatifactor*, and decreased the levels of opportunistic pathogenic bacteria (e.g., *Anaerofustis* and *Gammaproteobacteria*). This study demonstrates the utility of HFD and STZ induced rat models to analyze changes in the gut microbiota. Importantly, these findings highlight the potential importance of the gut microbiota on the hypoglycemic activities of GQD, presenting a new perspective on the mechanism of this traditional medicine, laying a foundation for further development in clinical application. Further work should focus on determining the best intervention time and most effective ingredients of treatment. Moreover, fecal microbiome transplantation can be performed to analyze the key pathways triggered by the treatment.

## Data Availability Statement

All the raw sequence data have been deposited in the Genome Sequence Archive (Wang Y. et al., 2017) in National Genomics Data Center (CNCB-NGDC Members and Partners, 2021), China National Center for Bioinformation / Beijing Institute of Genomics, Chinese Academy of Sciences, under accession number CRA004856 that are publicly accessible at https://ngdc.cncb.ac.cn/gsa.

## Ethics Statement

The animal study was reviewed and approved by Implementing Principles of Laboratory Animal Ethics of Guang’anmen Hospital, China Academy of Chinese Medical Sciences.

## Author Contributions

XT, ML, and JW conceived the original idea and designed the experiment. JT, ZG, YY, HW, and XW carried out the sample collection and data analysis. BB performed the bioinformatics analysis of the microbiota data. JT wrote the manuscript and BB reviewed the manuscript. All authors contributed to the article and approved the submitted version.

## Funding

This work was partially supported by the National Natural Science Foundation of China (No. 81430097, 81904187), Capital Health Development Research Project (CD2020-4-4155), and the Outstanding Young Scientific and Technological Talents Program (ZZ13-YQ-026). CACMS Scientific and Technological Innovation Fund, CI2021A01601; Open Project of National Facility for Translational Medicine (Shanghai), TMSK-2021-407.

## Conflict of Interest

The authors declare that the research was conducted in the absence of any commercial or financial relationships that could be construed as a potential conflict of interest.

## Publisher’s Note

All claims expressed in this article are solely those of the authors and do not necessarily represent those of their affiliated organizations, or those of the publisher, the editors and the reviewers. Any product that may be evaluated in this article, or claim that may be made by its manufacturer, is not guaranteed or endorsed by the publisher.

## Supplementary Material

Supplementary Figure 1 Diversity of the gut microbiota among different groups. **(A)** Number of genera. **(B)** Shannon diversity.

Supplementary Figure 2 Comparison of relative abundance between **(A)** DM0w vs. DM12w; **(B)** GQD0w vs. GQD12w; **(C)** Met0w vs. Met12w; **(D)** DM12w vs. NC12w; **(E)** GQD12w vs. DM12w; **(F)** Met12w vs. DM12w; **(G)** GQD12w vs. Met12w.

Supplementary Figure 3 Comparison of gut microbiota structure among NC, GQD and Met.
