# Supplementary figures and images for: Alleviation Effects of GQD, a Traditional Chinese Medicine Formula, on Diabetes Rats Linked to Modulation of the Gut Microbiome
Source: Front Cell Infect Microbiol. 2021 Oct 8;11:740236. doi: 10.3389/fcimb.2021.740236 (PMC8531589; doi:10.3389/fcimb.2021.740236)

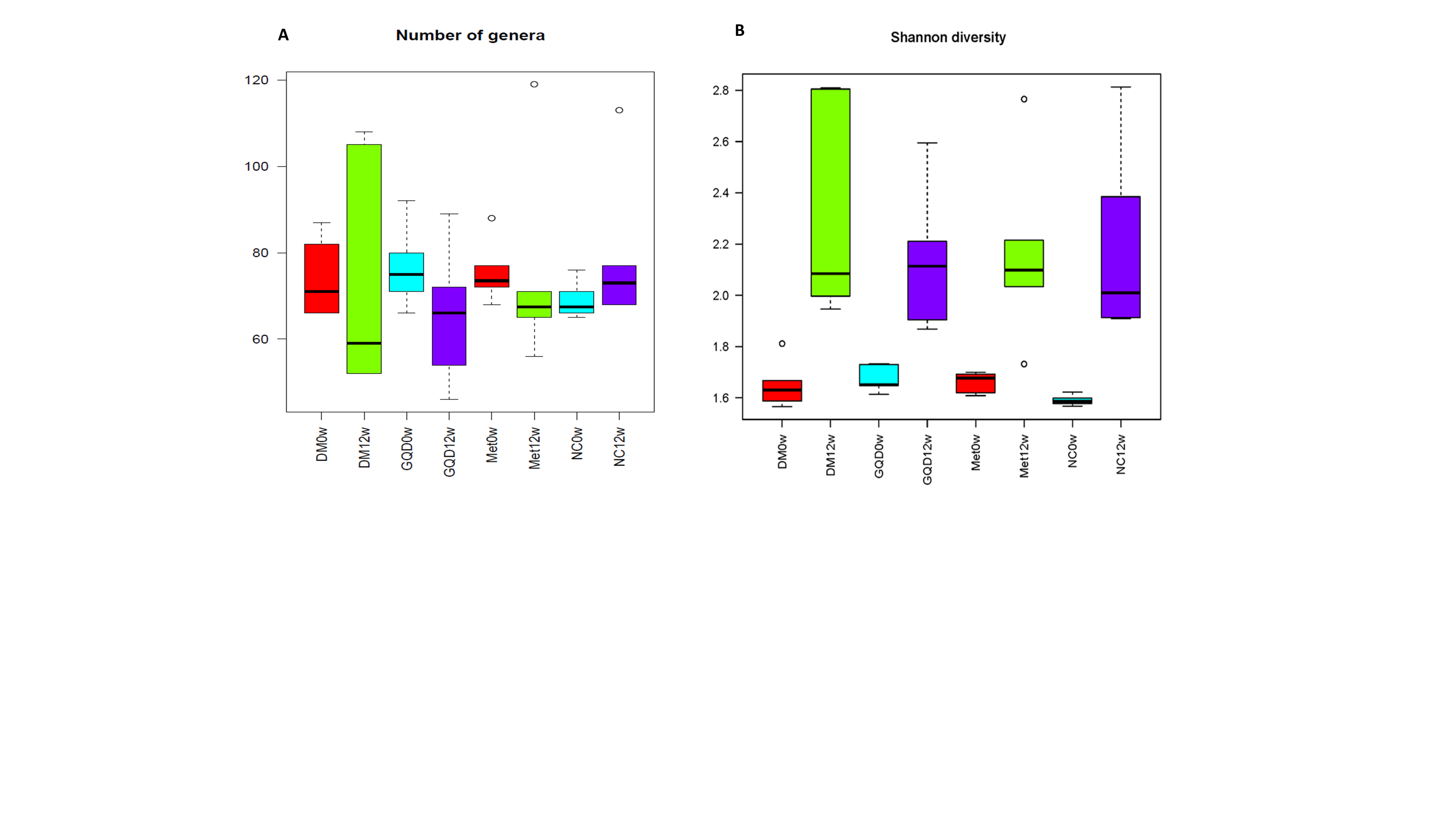

Supplement: Supplementary Figure 1 — Diversity of the gut microbiota among different groups. (A) Number of genera. (B) Shannon diversity. [file Image_1.tif]

A

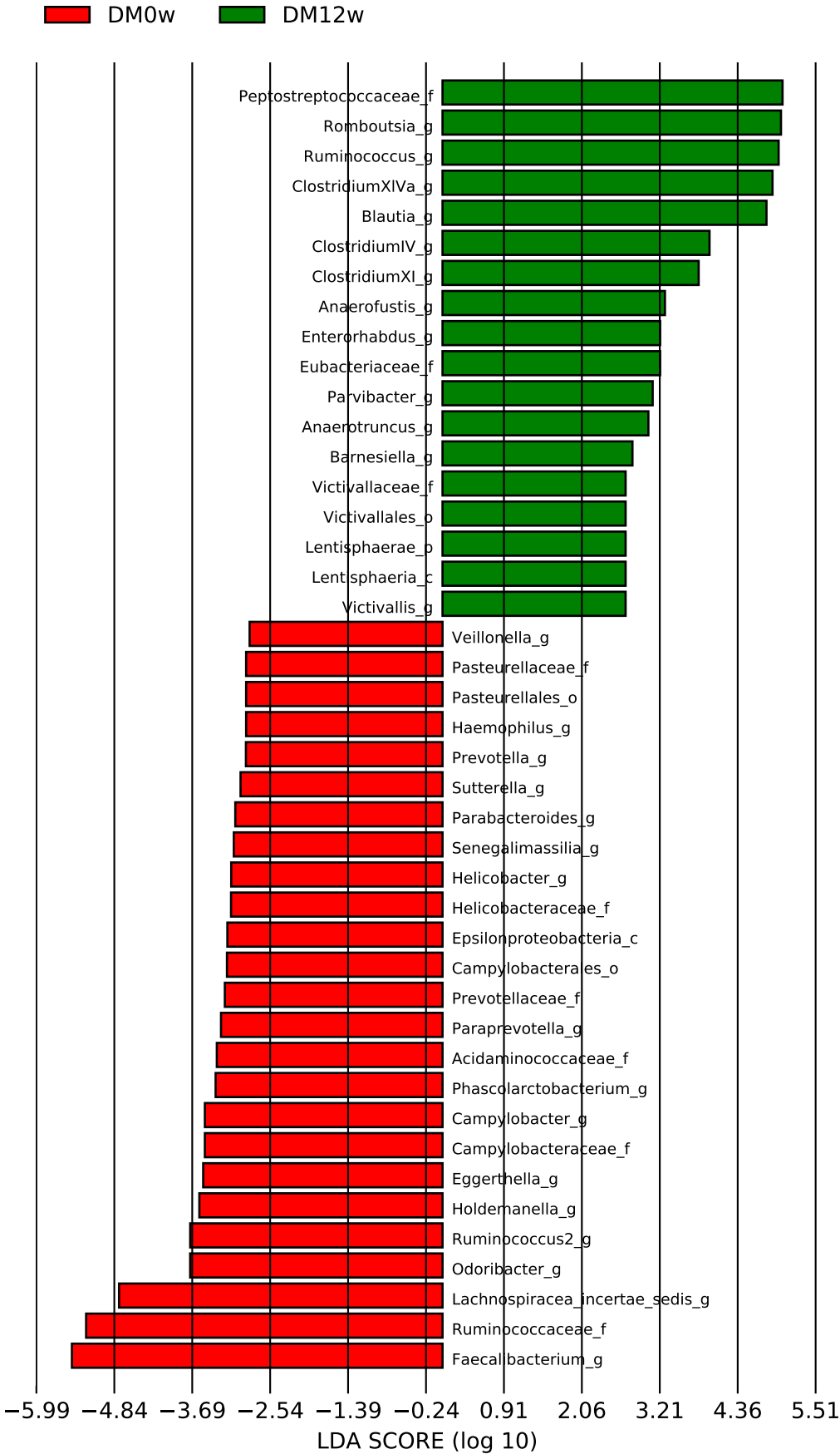

B

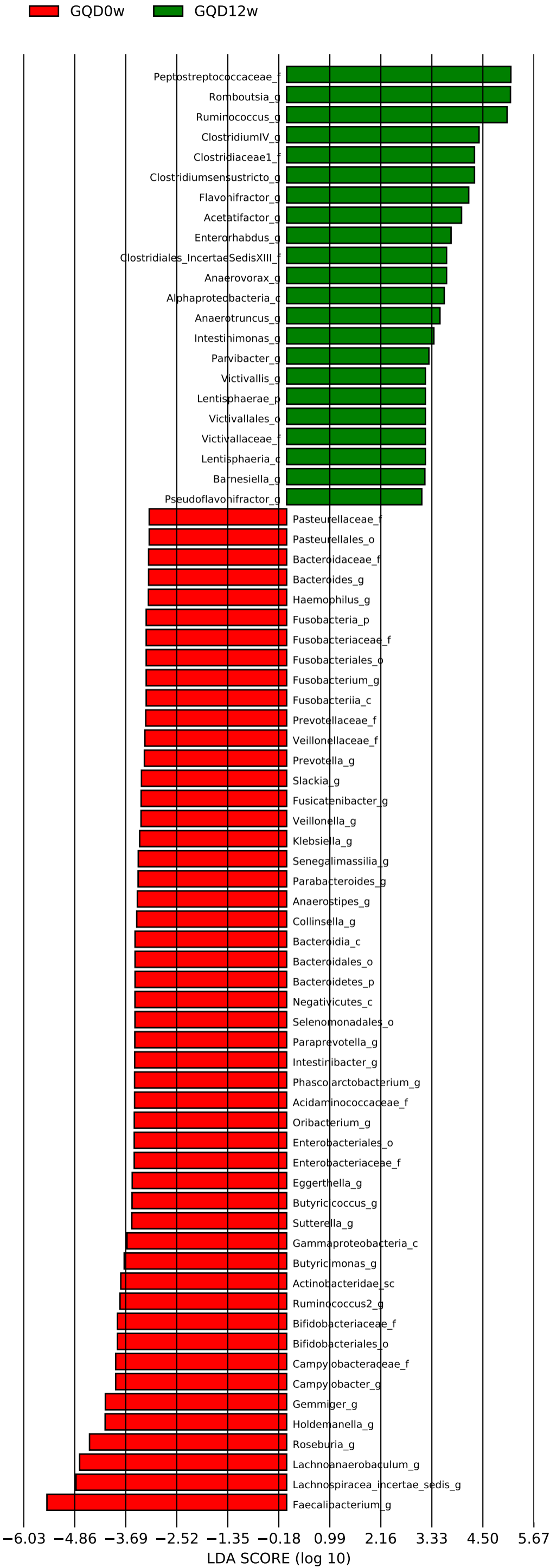

C

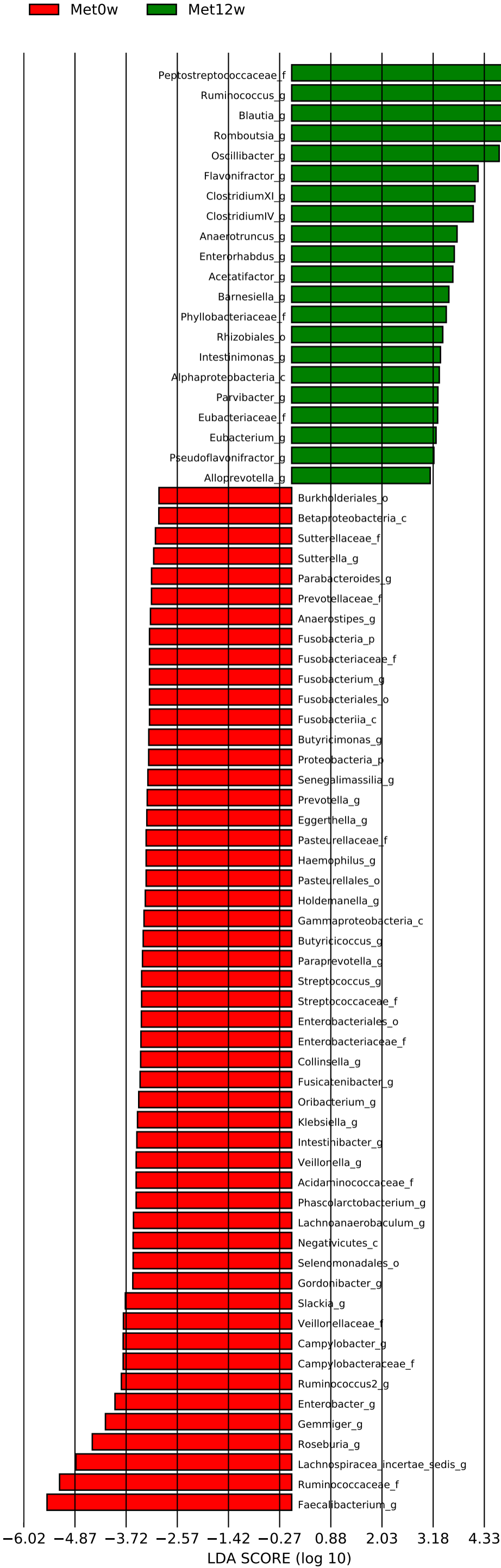

D

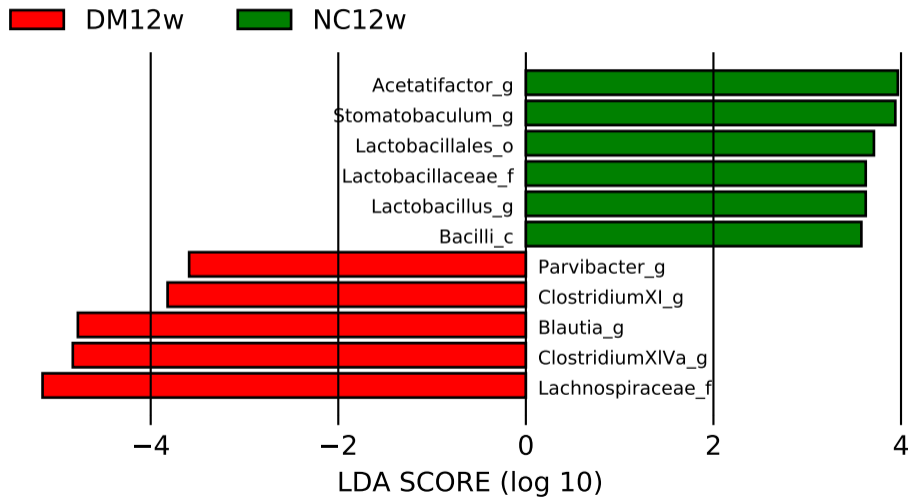

E

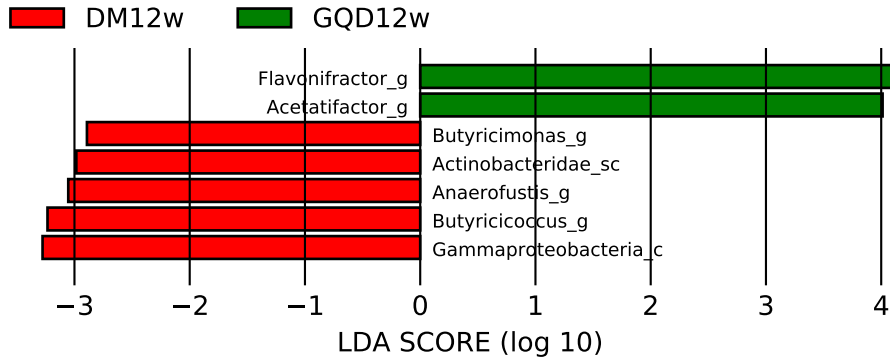

F

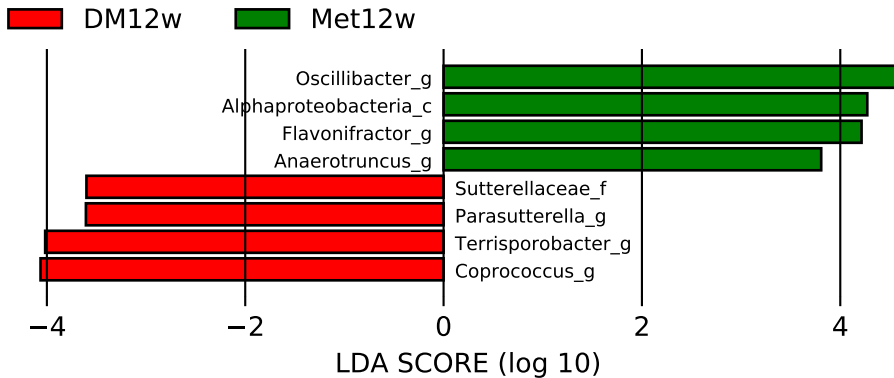

G

Met12w

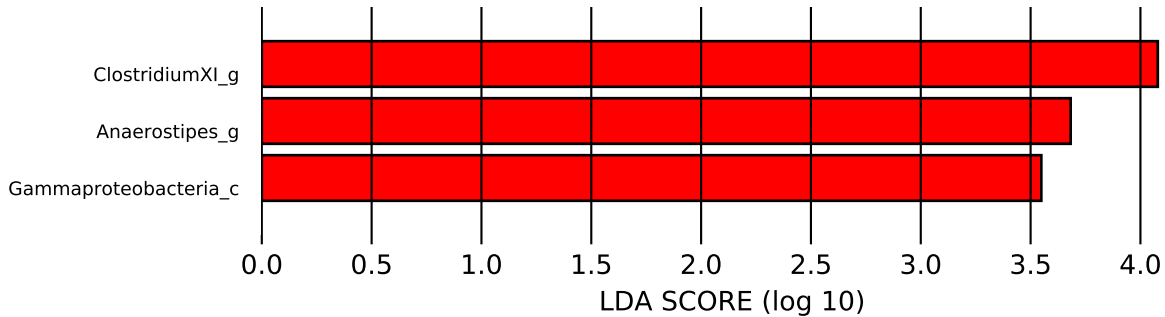

Supplement: Supplementary Figure 2 — Comparison of relative abundance between (A) DM0w vs. DM12w; (B) GQD0w vs. GQD12w; (C) Met0w vs. Met12w; (D) DM12w vs. NC12w; (E) GQD12w vs. DM12w; (F) Met12w vs. DM12w; (G) GQD12w vs. Met12w. [file Image_2.pdf]

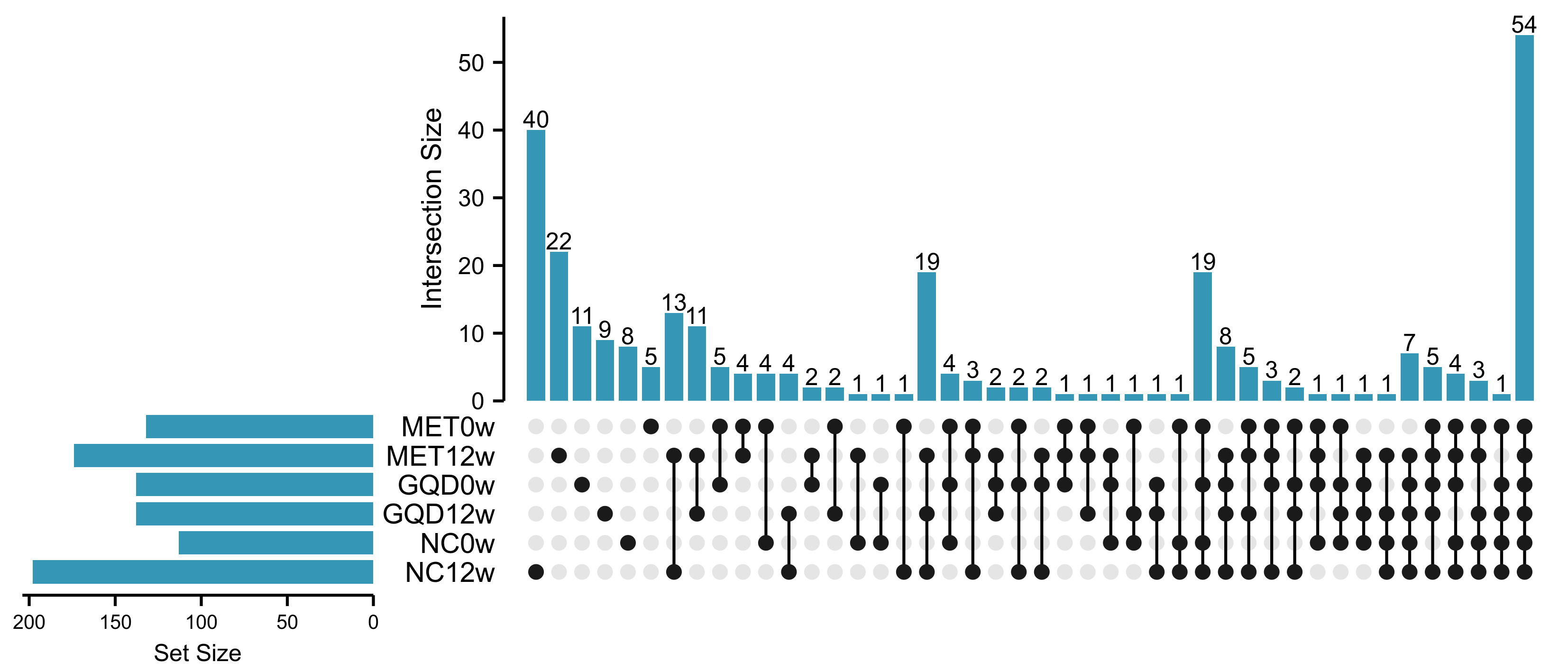

Supplement: Supplementary Figure 3 — Comparison of gut microbiota structure among NC, GQD and Met. [file Image_3.tiff]
